# Performance-Based Optimization of Soybean-Based Recycling Agents in SBS-Modified Asphalt Binders and High-RAP

**DOI:** 10.3390/polym18182289

**Published:** 2026-09-19

**Authors:** Anas AbuAlia, Ibrahim Elnaml, Louay N. Mohammad, Samuel B. Cooper, Gaylon L. Baumgardner

**Affiliations:** 1 Wisconsin Department of Transportation Northeast Region, Green Bay, WI 54304, USA; anas.abualia@dot.wi.gov; 2 Department of Civil and Environmental Engineering, Illinois Center of Transportation, University of Illinois Urbana-Champaign, 1611 Titan Dr, Rantoul, IL 61866, USA; elnaml@illinois.edu; 3 Department of Civil and Environmental Engineering, Louisiana Transportation Research Center, Louisiana State University, 4101 Gourrier Avenue, Baton Rouge, LA 70808, USA; 4 Louisiana Transportation and Research Center, Louisiana Department of Transportation and Development, 4101 Gourrier Ave, Baton Rouge, LA 70808, USA; samuel.cooperiii@la.gov; 5 Paragon Technical Services, Inc., Jackson, MS 39218, USA; gaylon.baumgardner@ergon.com

**Keywords:** reclaimed asphalt pavement, RAP, soybean-based recycling agents, asphalt mixtures, cracking resistance, permanent deformation, performance-based optimization, dosage range selection

## Abstract

Asphalt binders are complex viscoelastic polymeric materials whose rheological behavior is governed by interactions among their chemical constituents and, in polymer-modified systems, the morphology and stability of the dispersed polymer network. This study developed a performance-based framework for selecting the type and dosage of bio-based soybean recycling agents (RAs) for styrene-butadiene-styrene (SBS)-modified asphalt mixtures containing 30% reclaimed asphalt pavement (RAP). Ten dense-graded asphalt mixtures with a nominal maximum aggregate size of 12.5 mm were evaluated, including a control mixture produced with PG 76-22 (PG 67-22 asphalt binder modified with 3.5% SBS) and no RAP, and nine mixtures containing 30% RAP and three soybean-based recycling agents (RA1, RA2, and RA3) at dosages of 0.5%, 2.0%, and 4.0% by weight of binder. Binder characterization included Superpave performance grading, while mixture performance was evaluated using the Hamburg Wheel Tracking (HWT) test, freeze–thaw-conditioned HWT, Semi-Circular Bend (SCB), IDEAL-CT, IDEAL-RT, and Cantabro abrasion loss tests. Increasing recycling-agent dosage improved cracking resistance but progressively reduced rutting resistance, consistent with the measured decrease in binder viscosity. The magnitude of the softening effect followed the order RA3 > RA2 > RA1, consistent with measured viscosity reductions and HWT rut-depth response. Integrating SCB fracture resistance with the HWT rutting criterion identified acceptable dosage ranges of 1.4–4.0%, 0.5–2.8%, and 0.5–0.7% for RA1, RA2, and RA3, respectively. These results demonstrate that the effectiveness of the evaluated recycling agents, as reflected by binder rheology and asphalt mixture performance, influences the balance between fracture resistance and permanent deformation, providing a performance-based framework for optimizing bio-based recycling-agent selection in high-RAP polymer-modified asphalt mixtures.

## 1. Introduction

The use of reclaimed asphalt pavement (RAP) in asphalt mixtures has increased because it reduces virgin material demand, construction waste, and overall material cost [1,2,3]. However, RAP introduces an aged asphalt binder whose physicochemical properties have changed because of oxidative aging and alterations in binder composition [1,4,5]. Consequently, the aged binder is typically stiffer and more brittle than virgin binder. As RAP content increases, the contribution of the aged binder becomes more dominant, reducing workability and increasing cracking susceptibility unless the binder system is properly restored [1,4,6].

Styrene-butadiene-styrene (SBS) polymer modification is widely used to improve the rheological and mechanical performance of asphalt binders by enhancing elastic recovery and resistance to permanent deformation. When recycled materials are incorporated into SBS-modified mixtures, the aged RAP binder and recycling agents become part of the polymer-modified binder system, making the compatibility among these constituents an important factor governing the overall material response.

Recycling agents are commonly used to mitigate the effects of aged RAP binder by reducing binder stiffness and improving the functional response of recycled asphalt mixtures [4,5]. Their effectiveness depends on agent chemistry, dosage, diffusion, and compatibility with the aged binder [4,6,7]. Insufficient dosage may not provide adequate cracking resistance, whereas excessive dosage may over-soften the binder system and increase susceptibility to permanent deformation or moisture-related damage [8,9,10,11]. Therefore, recycling-agent dosage should be selected using mixture performance criteria rather than binder softening alone.

Soybean-based recycling agents have been used to reduce binder viscosity, improve blending and workability, and promote stress relaxation in RAP mixtures, thereby improving cracking resistance [4,8,9]. However, these benefits must be evaluated together with the potential reduction in permanent-deformation resistance associated with binder softening. This consideration is particularly important for mixtures containing high-RAP contents, where the interactions among the aged RAP binder, virgin binder, SBS-modified binder system, and the recycling agent govern the resulting mechanical performance.

Although previous studies have established the general benefits of recycling agents in RAP mixtures, fewer studies have directly compared multiple soybean-based recycling agents over several dosage levels within the same 30% RAP mixture framework while relating binder characterization to permanent deformation, moisture susceptibility, cracking resistance, and durability [4,6,7,8,9,10,11]. Limited information is also available on how differences in the softening efficiency of soybean-based recycling agents influence the balance between fracture resistance and rutting susceptibility under a constant RAP content and mixture structure. Therefore, this study evaluated three soybean-based recycling agents at three dosage levels in asphalt mixtures containing 30% RAP. The specific objective was to establish agent-specific acceptable dosage ranges that improve cracking resistance while maintaining acceptable permanent-deformation resistance using a performance-based selection framework.

The study was conducted within the Louisiana Department of Transportation and Development (DOTD) Balanced Mix Design (BMD) framework, which combines volumetric mixture design with performance verification of permanent deformation, intermediate-temperature cracking, durability, and moisture susceptibility. This framework provides a practical approach for evaluating the effectiveness of soybean-based recycling agents in SBS-modified, high-RAP asphalt mixtures. Within this framework, the present study develops a performance-based approach for selecting soybean-based recycling-agent types and dosages by integrating binder characterization with balanced mixture performance testing in 30% RAP asphalt mixtures.

## 2. Objective and Scope

The objective of this study was to evaluate and compare the effects of soybean-based recycling-agent types and dosages on the performance of SBS-modified asphalt mixtures incorporating 30% reclaimed asphalt pavement (RAP). The study further aimed to establish agent-specific dosage ranges that improve cracking resistance while maintaining acceptable permanent-deformation resistance through a performance-based evaluation framework.

A total of ten asphalt mixtures were evaluated, including one control mixture containing a PG 76-22 asphalt binder and no RAP, and nine mixtures incorporating 30% RAP, prepared using a PG 67-22 base asphalt binder modified with three soybean-based recycling agents, designated RA1, RA2, and RA3, at dosages of 0.5%, 2.0%, and 4.0% by weight of binder. The control binder was modified with 3.5% styrene-butadiene-styrene (SBS) polymer by weight of binder to produce the PG 76-22 binder.

A dense-graded asphalt mixture with a 12.5 mm nominal maximum aggregate size (NMAS) was designed in accordance with the Louisiana DOTD mix design specifications [12]. The control mixture contained 5.4% asphalt binder by total mixture weight, whereas the 30% RAP mixtures contained 5.5% asphalt binder. Aggregate gradation and volumetric properties satisfied the Louisiana DOTD specification requirements.

**Figure 1:**
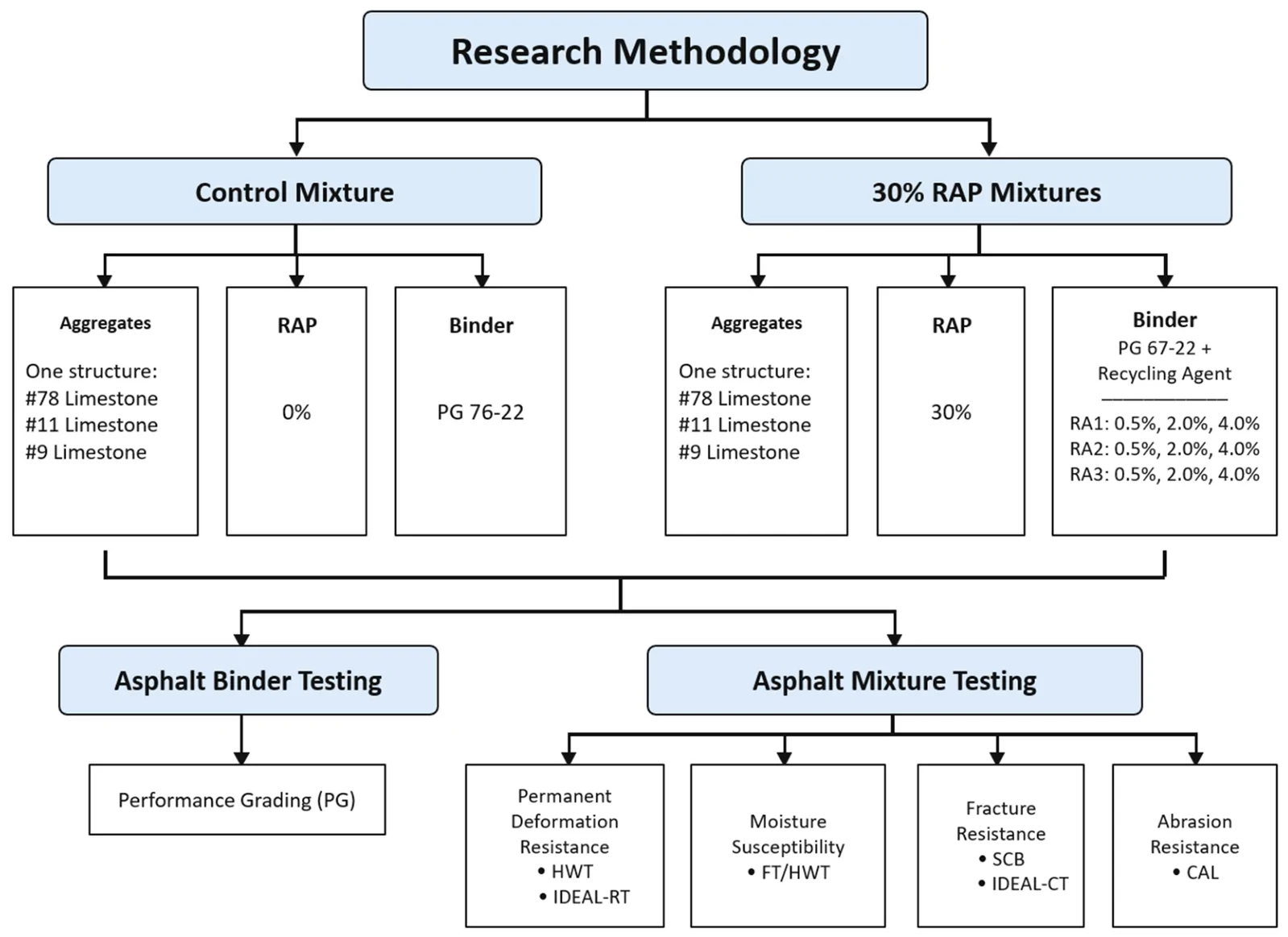
Research Methodology. Note: RAP = reclaimed asphalt pavement; NMAS = nominal maximum aggregate size; SBS = styrene-butadiene-styrene; RA1, RA2, and RA3 = soybean-based recycling-agent types; PG = performance grade; HWT = Hamburg Wheel Tracking test; IDEAL-RT = IDEAL rutting test; FT = freeze–thaw conditioning; SCB = Semi-Circular Bend test; IDEAL-CT = IDEAL cracking test; CAL = Cantabro abrasion loss test.

The asphalt binder characterization consisted of Superpave performance grading to quantify the influence of the soybean-based recycling agents on binder rheological properties. Asphalt mixture performance was evaluated using the Hamburg Wheel Tracking (HWT) test for permanent-deformation resistance, freeze–thaw-conditioned HWT for moisture susceptibility, Semi-Circular Bend (SCB) and IDEAL-CT tests for cracking resistance, IDEAL-RT for rutting tolerance, and the Cantabro abrasion loss test for durability. Three replicate specimens were tested for each mixture except for the HWT and SCB tests, where four specimens were evaluated. The overall experimental methodology is illustrated in Figure 1.

## 3. Experimental Design

The following sections describe the constituent materials, asphalt mixture design, specimen preparation, and the laboratory testing program used to evaluate the influence of soybean-based recycling-agent types and dosages on the rheological properties of SBS-modified asphalt binders and the performance of asphalt mixtures containing 30% reclaimed asphalt pavement (RAP).

### 3.1. Materials

#### 3.1.1. Asphalt Binder

Ten asphalt binders were evaluated in this study, as summarized in Table 1. Binder B1 was a PG 76-22 SBS-modified asphalt binder containing 3.5% styrene-butadiene-styrene (SBS) polymer by weight of binder and served as the control binder. Binders B2 through B10 were prepared using a PG 67-22 base asphalt binder modified with three soybean-based recycling agents, designated RA1, RA2, and RA3, at dosages of 0.5%, 2.0%, and 4.0% by weight of binder.

Table 2 summarizes the physical properties of the three soybean-based recycling agents. The recycling agents differed primarily in kinematic viscosity while maintaining relatively high flash points, indicating differences in their softening capability while preserving acceptable handling characteristics for asphalt-binder modification.

Table 3 presents the asphalt-binder performance grading results according to AASHTO M 320 [15] and the Louisiana Standard Specifications for Roads and Bridges [12].

#### 3.1.2. Aggregates

Three aggregate sources commonly used in Louisiana were incorporated into the study: #78 limestone, #11 limestone, and #9 limestone. All aggregates satisfied the Louisiana DOTD requirements for 12.5 mm nominal maximum aggregate size (NMAS) mixtures. The required aggregate properties are summarized below [12]:

- Flat and elongated particles, 5:1 ratio, maximum = 10%
- Coarse aggregate angularity, double-faced crushed particles, minimum = 90%
- Fine aggregate angularity, minimum = 45%
- Sand equivalent, minimum = 40%

### 3.2. Asphalt Mixture Design

Ten dense-graded asphalt mixtures with a 12.5 mm nominal maximum aggregate size (NMAS) were designed for traffic levels exceeding 3 million equivalent single-axle loads (ESALs) in accordance with AASHTO R35 [16] and the Louisiana DOTD specifications [12]. The control mixture contained a PG 76-22 SBS-modified asphalt binder and no RAP, whereas the remaining mixtures contained 30% RAP and PG 67-22 asphalt binder modified with soybean-based recycling agents. All mixtures were designed to satisfy the DOTD gradation and volumetric requirements.

Table 4 presents the job mix formulas for the control and 30% RAP mixtures. The control mixture, prepared with the PG 67-22 asphalt binder modified with 3.5% SBS, had an asphalt binder content of 5.4% by total mixture weight, while the 30% RAP mixtures had an asphalt binder content of 5.5%. The RAP material contained 5.2% asphalt binder. The mixing process started by adding 5.0% moisture to the RAP material and storing it overnight at room temperature. The virgin aggregates were superheated to 195 °C and mixed with RAP for 4 min. The blended RAP and aggregate mixture was then placed in an oven at 163 °C until the target mixing temperature was reached. Subsequently, the asphalt binder, preheated to 163 °C, was added and mixing continued for an additional 4 min [17]. The mixtures were then short-term aged for 2 h at 163 °C and compacted using a Superpave gyratory compactor to the dimensions required for each test. All soybean-based recycling agents were preblended with the PG 67-22 base asphalt binder by the manufacturer before mixture production.

The baseline mixture (B1) represents the conventional Louisiana DOTD mixture, whereas mixtures B2–B10 represent 30% RAP alternatives incorporating soybean-based recycling agents at different dosages. Comparisons with the baseline mixture are designed to evaluate whether these practical high-RAP mixtures can achieve balanced performance comparable to the current Louisiana DOTD practice.

### 3.3. Testing Methods

Table 5 summarizes the laboratory testing program. Specimens were mixed and compacted using a Superpave gyratory compactor to achieve a target air-void content of 3.5 ± 0.5%. HWT and IDEAL-RT tests were conducted on short-term aged (STA) specimens to evaluate their permanent-deformation resistance and rutting tolerance, respectively. Freeze–thaw-conditioned HWT testing was performed to assess moisture susceptibility. SCB testing was conducted on both STA and long-term aged (LTA) specimens to distinguish the immediate influence of recycling-agent softening from the cracking response following extended laboratory aging. Long-term aging was performed for 120 h at 85 °C in accordance with AASHTO R30 [18]. Cantabro abrasion loss and IDEAL-CT tests were conducted on LTA-conditioned specimens. Three replicate specimens were tested for each mixture except for the HWT and SCB tests, for which four specimens were evaluated.

## 4. Results and Discussion

This section presents the mechanical performance results of the evaluated asphalt mixtures. Statistical analysis of variance (ANOVA) with Scheffe’s multiple-comparison procedure at a 95% confidence level was performed using SPSS Version 23. In the figures, letter groupings indicate statistical rankings, where A represents the best-performing group and shared letters indicate no statistically significant difference between mixtures. Error bars represent the 95% confidence interval of the mean [25].

### 4.1. Asphalt Mixture Performance

The performance evaluation considered permanent-deformation resistance, moisture susceptibility, cracking resistance, abrasion resistance, and rutting tolerance. The results are discussed according to the governing distress mechanism to evaluate the influence of soybean-based recycling-agent type and dosage on the performance of 30% RAP asphalt mixtures and to establish acceptable dosage ranges.

#### 4.1.1. Permanent Deformation

**Figure 2:**
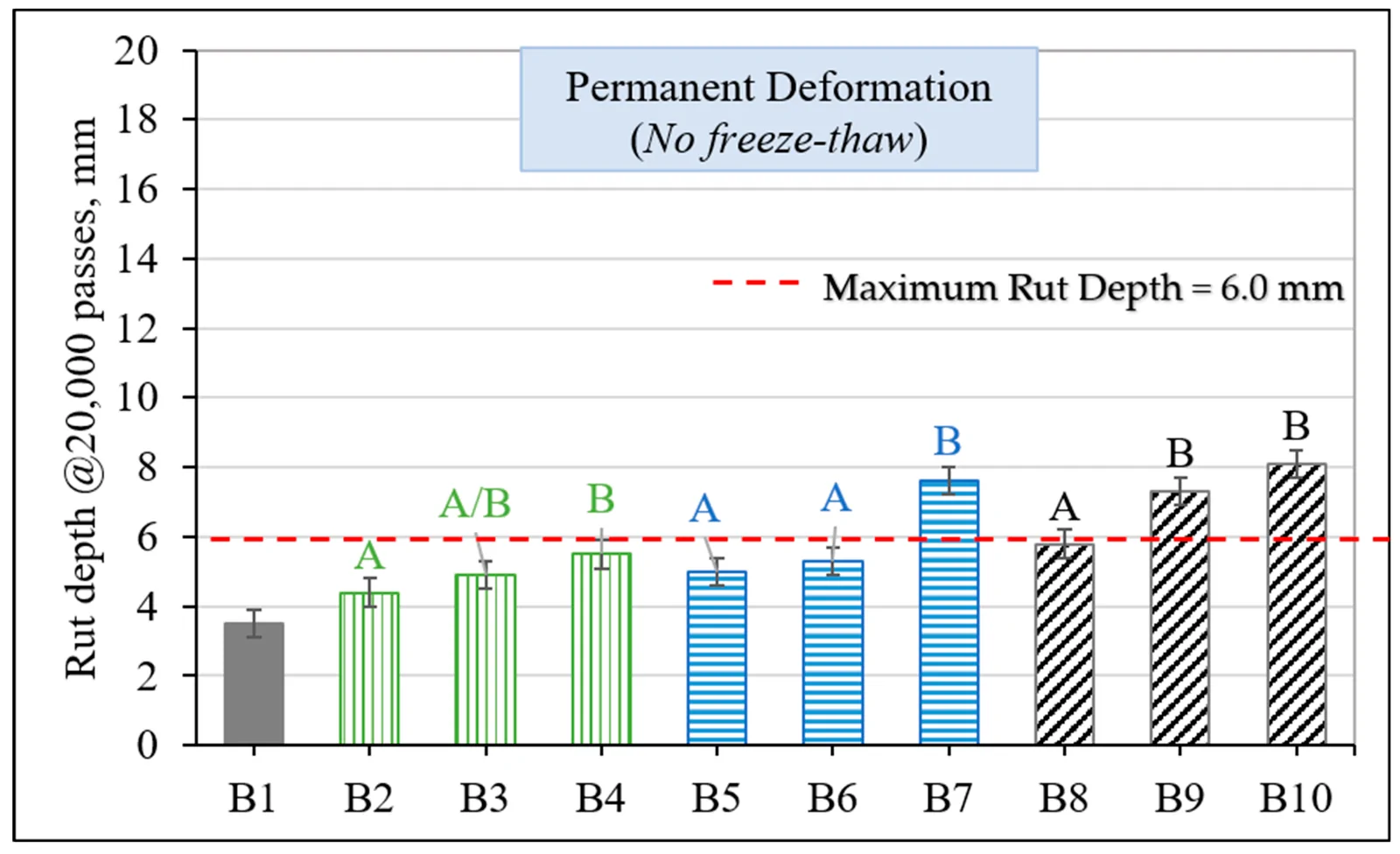
HWT test results—Permanent deformation. Notes: Green letters represent RA1 statistical group, blue letters represent RA2 statistical group, and black letters represent RA3 statistical group.

Figure 2 presents the Hamburg Wheel Tracking (HWT) rut depths of the unconditioned mixtures after 20,000 wheel passes. The coefficient of variation (CoV) ranged from 7% to 14%, with an average of 10.6%, indicating good testing repeatability. Lower rut depth corresponds to greater resistance to permanent deformation. The Louisiana DOTD Balanced Mix Design (BMD) specification establishes a maximum allowable rut depth of 6.0 mm after 20,000 passes [12].

All mixtures satisfied the DOTD rut-depth criterion except B7, B9, and B10, Figure 2. Consequently, the HWT results establish the upper acceptable dosage limit for each recycling agent by identifying the dosage at which binder softening begins to compromise permanent-deformation resistance.

The control mixture (B1), prepared with the SBS-modified PG 76-22 binder, exhibited the lowest rut depth and therefore the greatest resistance to permanent deformation. The incorporation of soybean-based recycling agents progressively increased rut depth as the dosage increased, indicating that the reduction in binder viscosity improved mixture flexibility but reduced high-temperature stiffness. The magnitude of this softening effect followed the order RA3 > RA2 > RA1. For example, mixture B9 containing 2.0% RA3 exhibited a rut depth of 7.3 mm compared with 4.9 mm for B3 (2.0% RA1) and 5.3 mm for B6 (2.0% RA2). This RA ranking was inferred from binder rheological characterization and asphalt mixture performance testing as detailed chemical characterization (i.e., Fourier-transform infrared spectroscopy (FTIR) or compositional analysis) was beyond the scope of this study.

This trend is consistent with the binder characterization results presented in Table 2 and Table 3. RA3 exhibited the lowest kinematic viscosity among the evaluated recycling agents and produced the greatest reduction in binder performance grade, whereas RA1 exhibited the highest viscosity and the smallest reduction in binder stiffness. The corresponding HWT results demonstrate that the rheological changes introduced by the recycling agents were directly reflected in mixture permanent-deformation resistance. These findings indicate that recycling-agent viscosity and the resulting modification of the SBS-modified binder system govern the rutting susceptibility of the evaluated mixtures. Thus, the HWT rut-depth criterion was selected as the primary permanent-deformation performance criterion for establishing acceptable recycling-agent dosage ranges.

**Figure 3:**
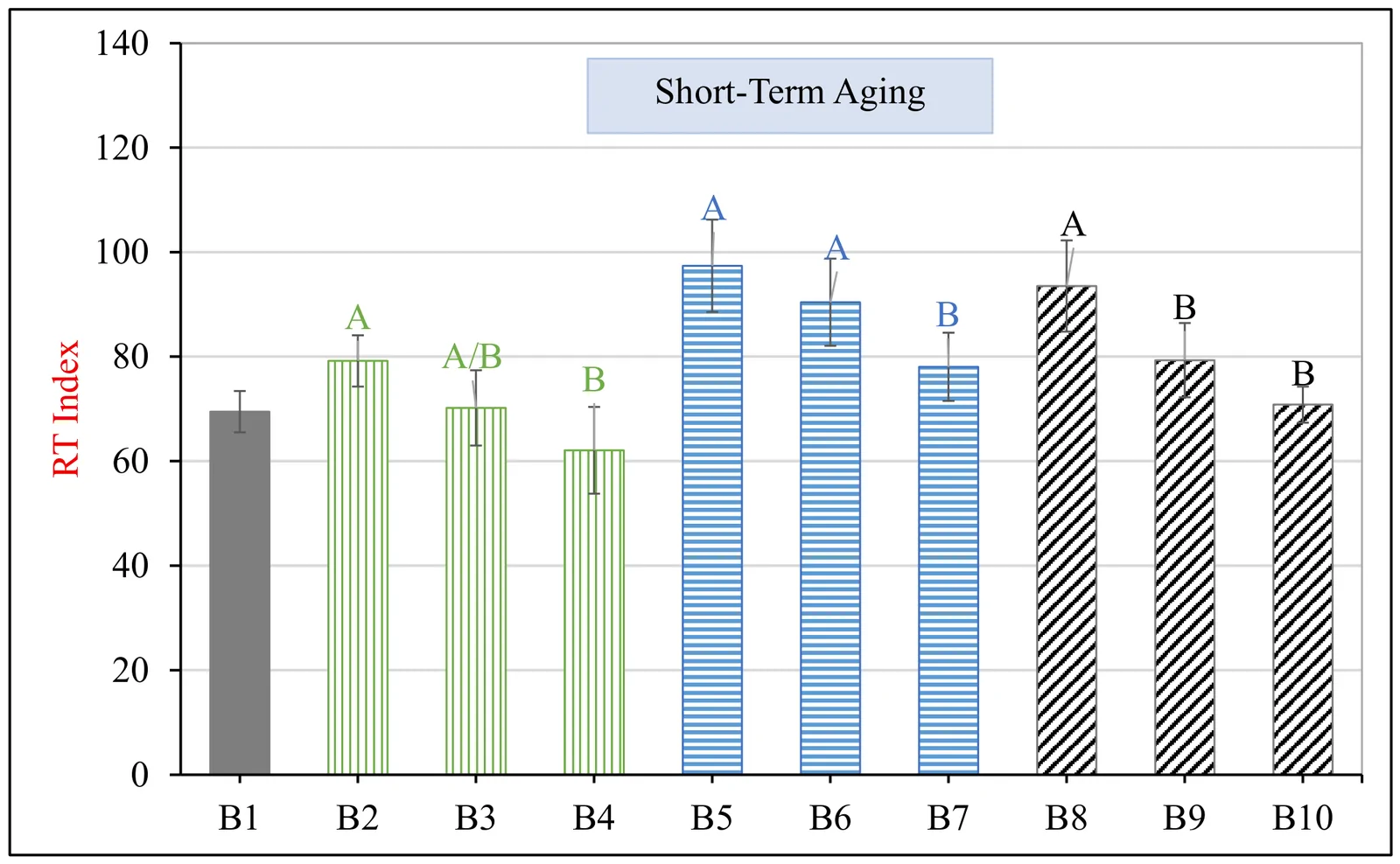
IDEAL-RT test results—Short-term aging. Notes: Green letters represent RA1 statistical group, blue letters represent RA2 statistical group, and black letters represent RA3 statistical group.

Figure 3 presents the IDEAL-RT results for the evaluated mixtures after short-term aging (STA). The coefficient of variation (CoV) ranged from 5% to 13%, with an average of 8.5%, indicating good testing repeatability. Higher RT index values indicate greater resistance to permanent deformation. Although ASTM D8360 does not currently specify an acceptance criterion for the RT index, NCHRP Project 20-44 [26] recommends a minimum RT index of 75 for mixtures containing PG 76-xx binders.

The RT index generally decreased with increasing recycling-agent dosage, indicating that greater binder softening reduced rutting tolerance. This trend is consistent with the binder characterization results (Table 2 and Table 3), which showed progressive reductions in binder viscosity and high-temperature performance grade as recycling-agent dosage increased.

Unlike the HWT results, however, the IDEAL-RT test did not consistently differentiate the relative rutting performance of the evaluated mixtures. Several 30% RAP mixtures exhibited RT index values comparable to or greater than those of the control mixture despite exhibiting higher rut depths in the HWT test. For example, mixtures containing RA2 and RA3 at lower dosages achieved RT index values exceeding those of the control mixture, whereas their HWT results indicated greater susceptibility to permanent deformation. This difference suggests that the IDEAL-RT test was less sensitive than the HWT test to the changes in high-temperature mixture performance produced by the soybean-based recycling agents. Consequently, IDEAL-RT was used as a complementary indicator of rutting tolerance, whereas the HWT test was adopted as the primary permanent-deformation criterion because it directly measures wheel-tracking susceptibility and is consistent with the Louisiana DOTD Balanced Mix Design (BMD) rut-depth requirement used to establish the acceptable recycling-agent dosage ranges.

#### 4.1.2. Moisture Susceptibility

**Figure 4:**
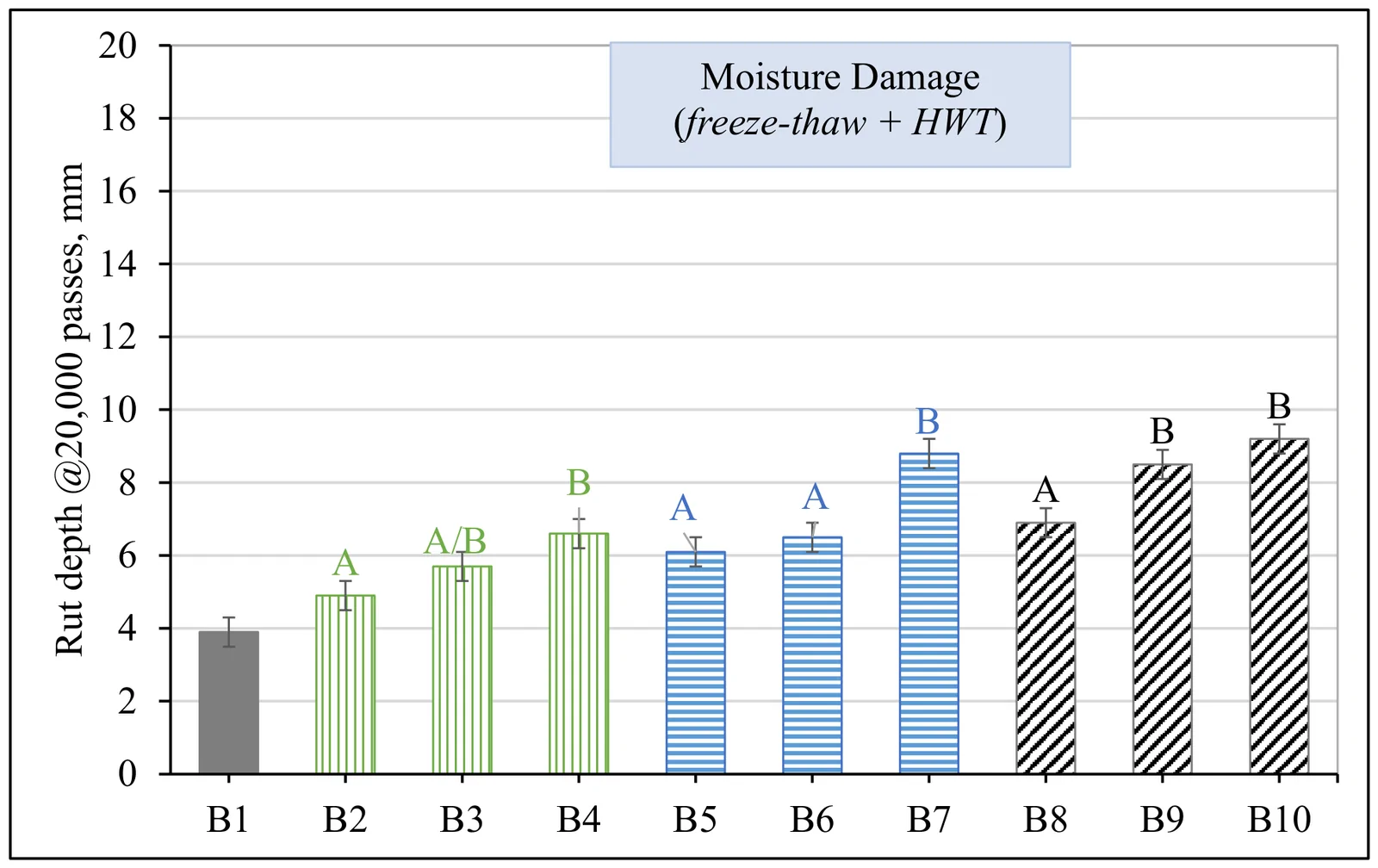
HWT test results—Freeze–thaw. Notes: Green letters represent RA1 statistical group, blue letters represent RA2 statistical group, and black letters represent RA3 statistical group.

Figure 4 presents the Hamburg Wheel Tracking (HWT) rut depths of the mixtures after one freeze–thaw conditioning cycle and 20,000 wheel passes. The coefficient of variation (CoV) ranged from 9% to 16%, with an average of 12.6%, indicating acceptable testing repeatability. A lower rut depth after freeze–thaw conditioning corresponds to lower moisture susceptibility. Because the Louisiana DOTD Balanced Mix Design (BMD) specification does not establish a separate acceptance criterion for freeze–thaw-conditioned HWT testing, the results were used to compare the moisture susceptibility of the evaluated mixtures rather than to establish pass/fail criteria.

During the freezing cycle, water expands and exerts pressure forces on the internal structure of the asphalt mixture. This expansion leads to both an increase in the size of individual air voids and the coalescence of adjacent voids, which facilitates the formation of new air voids. Subsequently, during the thawing cycle, water infiltrates and fills these newly formed and enlarged voids, severely compromising the internal structural integrity of the asphalt mixture [27,28].

Freeze–thaw conditioning increased the HWT rut depth of all mixtures by 11.4% to 22.6%, indicating that moisture conditioning reduced resistance to wheel-tracking deformation. The freeze–thaw-conditioned HWT results produced the same ranking of recycling-agent performance as the standard HWT results, indicating that moisture conditioning increased rut depth without changing the relative influence of recycling-agent type. Rut-depth susceptibility followed the order RA3 > RA2 > RA1, demonstrating that the relative effectiveness of the recycling agents remained unchanged after moisture conditioning. This trend is consistent with the binder characterization results (Table 2 and Table 3), where RA3 produced the greatest reduction in binder viscosity and high-temperature performance grade, while RA1 exhibited the smallest softening effect.

These results suggest that the increased rut depths following freeze–thaw conditioning were primarily associated with reduced resistance to permanent deformation rather than moisture-induced stripping.

Although freeze–thaw conditioning increased rut-depth susceptibility for all mixtures, the relative performance ranking remained unchanged. Consequently, the freeze–thaw-conditioned HWT test was used to compare the moisture susceptibility of the evaluated recycling agents but was not incorporated into the dosage-selection framework because no acceptance criterion currently exists in the Louisiana DOTD Balanced Mix Design (BMD) specification.

#### 4.1.3. Fracture Resistance

**Figure 5:**
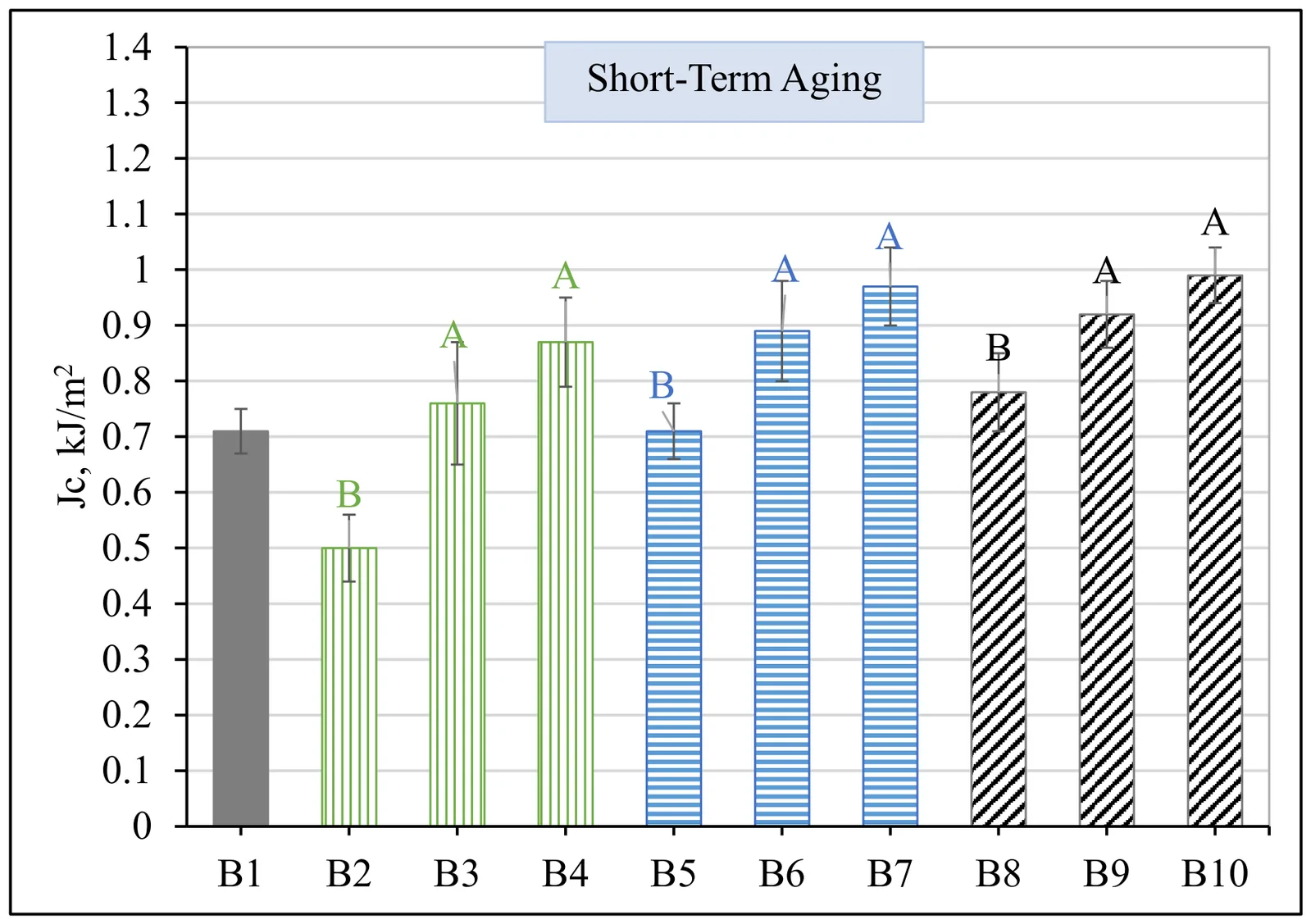
SCB test results—Short-term aging. Notes: Green letters represent RA1 statistical group, blue letters represent RA2 statistical group, and black letters represent RA3 statistical group.

**Figure 6:**
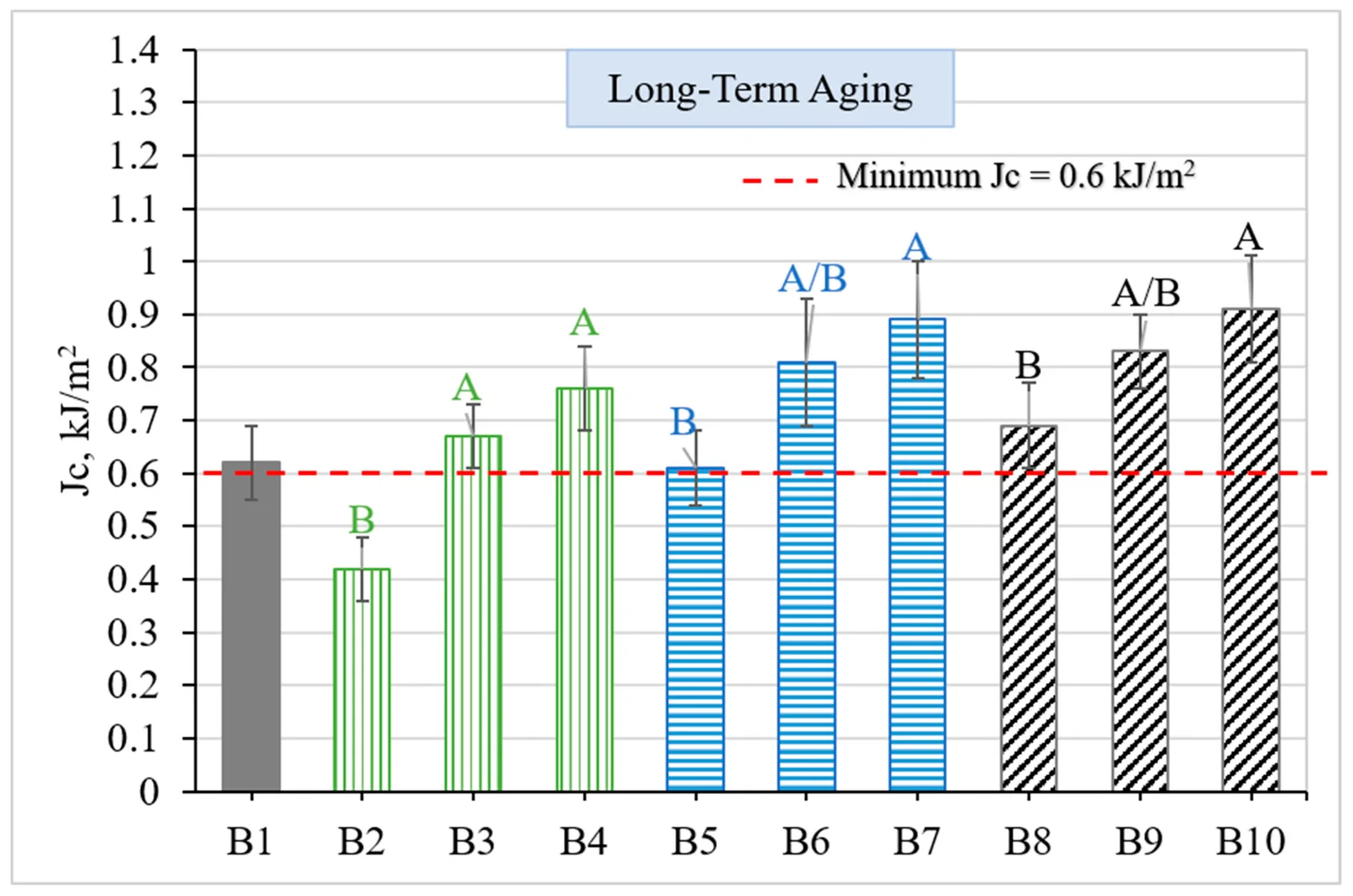
SCB test results—Long-term aging. Notes: Green letters represent RA1 statistical group, blue letters represent RA2 statistical group, and black letters represent RA3 statistical group.

Figure 5 and Figure 6 present the Semi-Circular Bend (SCB) critical strain energy release rate (Jc) results for the evaluated mixtures after short-term aging (STA) and long-term aging (LTA), respectively. The coefficient of variation (CoV) ranged from 6% to 13%, with an average of 9.2%, indicating good testing repeatability. Higher Jc values indicate greater resistance to intermediate-temperature cracking. The Louisiana DOTD Balanced Mix Design (BMD) specification requires a minimum Jc of 0.6 kJ/m^2^ for long-term aged mixtures [12].

The STA results, as seen in Figure 5, demonstrate that increasing recycling-agent dosage generally increased Jc, indicating improved fracture resistance immediately after mixture production. This trend became more evident after long-term aging, as seen in Figure 6, confirming that recycling-agent type and dosage significantly influenced the retained cracking resistance of the 30% RAP mixtures.

The control mixture (B1) exhibited a long-term aged Jc value of 0.62 kJ/m^2^, satisfying the Louisiana DOTD minimum requirement. In contrast, mixture B2 containing 0.5% RA1 produced a Jc value of 0.42 kJ/m^2^, indicating that this dosage was insufficient to compensate for the stiffening effect of the aged RAP binder. Increasing the RA1 dosage to 2.0% and 4.0% increased Jc above the specification threshold, demonstrating that additional recycling agent was required to restore adequate fracture resistance.

The recycling agents differed in their effectiveness. Mixtures containing 0.5% RA2 (B5) and 0.5% RA3 (B8) satisfied the fracture-resistance criterion, indicating that these recycling agents provided greater crack-resistance improvement at lower dosage levels than RA1. As dosage increased, Jc continued to increase for all three recycling agents, demonstrating that greater binder softening enhanced the ability of the mixtures to dissipate fracture energy.

The fracture-resistance trends are consistent with the binder characterization results, Table 2 and Table 3. Recycling agents that produced greater reductions in binder viscosity also produced larger increases in Jc, indicating that modification of the SBS-modified binder system improved stress relaxation and reduced mixture brittleness. However, the improved cracking resistance must be considered together with the corresponding reduction in permanent-deformation resistance discussed in Section 4.1.1 and Section 4.1.2.

It is noted that, consistent with the objective of this study to establish acceptable recycling-agent dosage ranges, the long-term aged SCB Jc criterion was adopted as the lower performance bound, while the HWT rut-depth criterion defined the upper performance bound. The intersection of these two criteria formed the basis of the performance-based dosage-selection framework developed in this study.

**Figure 7:**
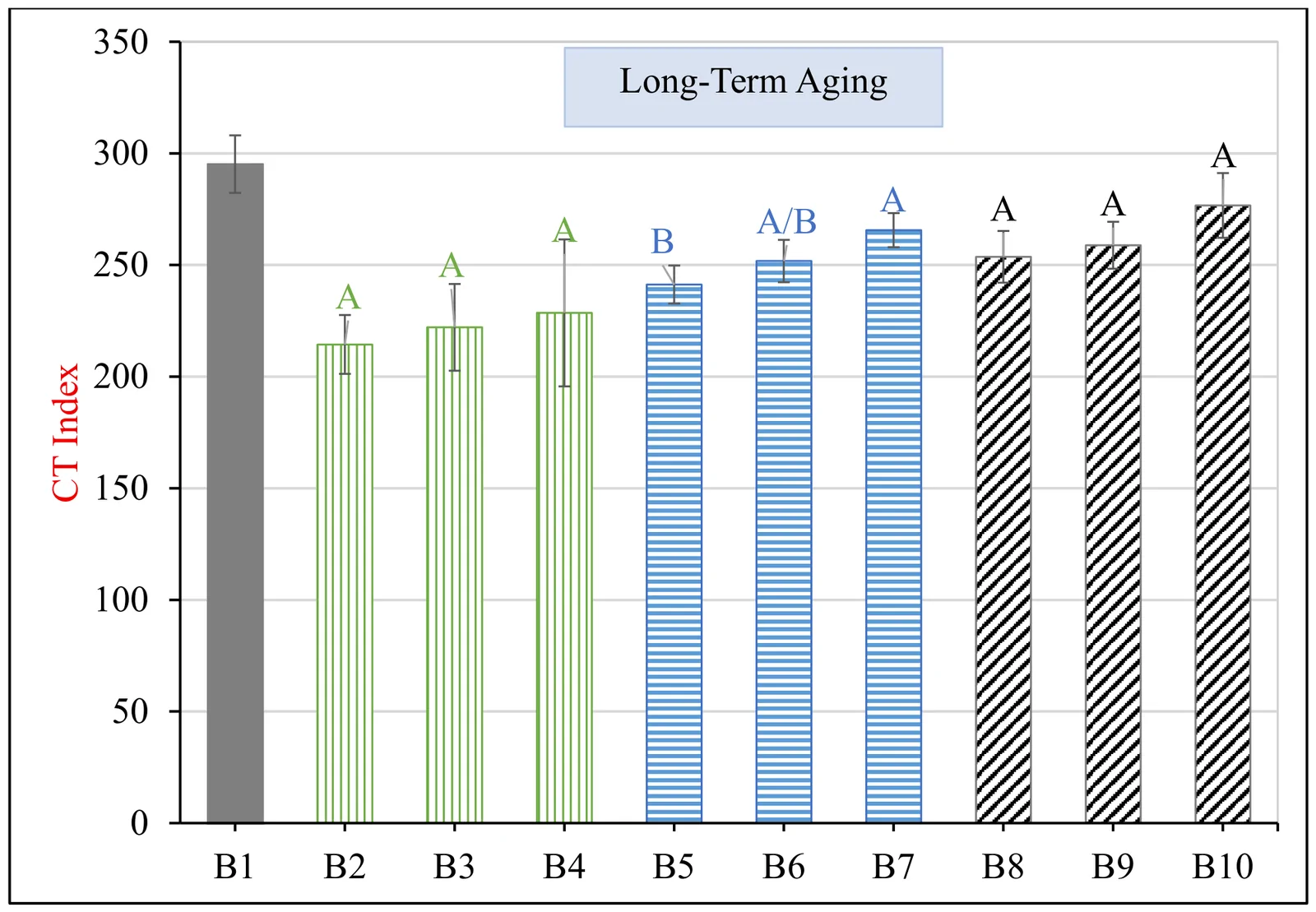
IDEAL-CT Test Results—Long-Term Aging. Notes: Green letters represent RA1 statistical group, blue letters represent RA2 statistical group, and black letters represent RA3 statistical group.

Figure 7 presents the IDEAL-CT results for the evaluated mixtures after long-term aging (LTA). The coefficient of variation (CoV) ranged from 3.0% to 14.4%, with an average of 5.8%, indicating excellent testing repeatability. Higher CT index values indicate greater cracking tolerance. Although ASTM D8225 does not currently specify an acceptance criterion for the CT index, the NCHRP Project 20-44 [26] recommends minimum CT index values of 90 and 40 for short-term aged (STA) and intermediate-aged mixtures, respectively.

Increasing the recycling-agent dosage generally increased the CT index for all three soybean-based recycling agents, indicating improved cracking tolerance after long-term aging. Similarly to the SCB results shown in Figure 5 and Figure 6, the improvement became more pronounced as the recycling-agent dosage increased, demonstrating that greater binder softening enhanced the resistance of the 30% RAP mixtures to crack initiation and propagation.

Although the IDEAL-CT results followed the same overall trend as the SCB Jc results, they provided less discrimination among the evaluated mixtures. In particular, mixtures containing RA1 and RA3 exhibited fewer statistically significant differences over the evaluated dosage range, whereas the SCB Jc test more clearly distinguished the influence of recycling-agent types and dosages on fracture resistance. Consequently, the IDEAL-CT test confirmed the overall cracking trends but provided less sensitivity for identifying the optimum recycling-agent dosage.

Accordingly, IDEAL-CT was used as a complementary indicator of cracking tolerance, whereas the long-term aged SCB Jc criterion was adopted as the primary cracking-performance criterion because it provided greater sensitivity for differentiating recycling-agent effectiveness and directly satisfied the Louisiana DOTD Balanced Mix Design (BMD) specification used in the performance-based dosage-selection framework.

#### 4.1.4. Abrasion Resistance

**Figure 8:**
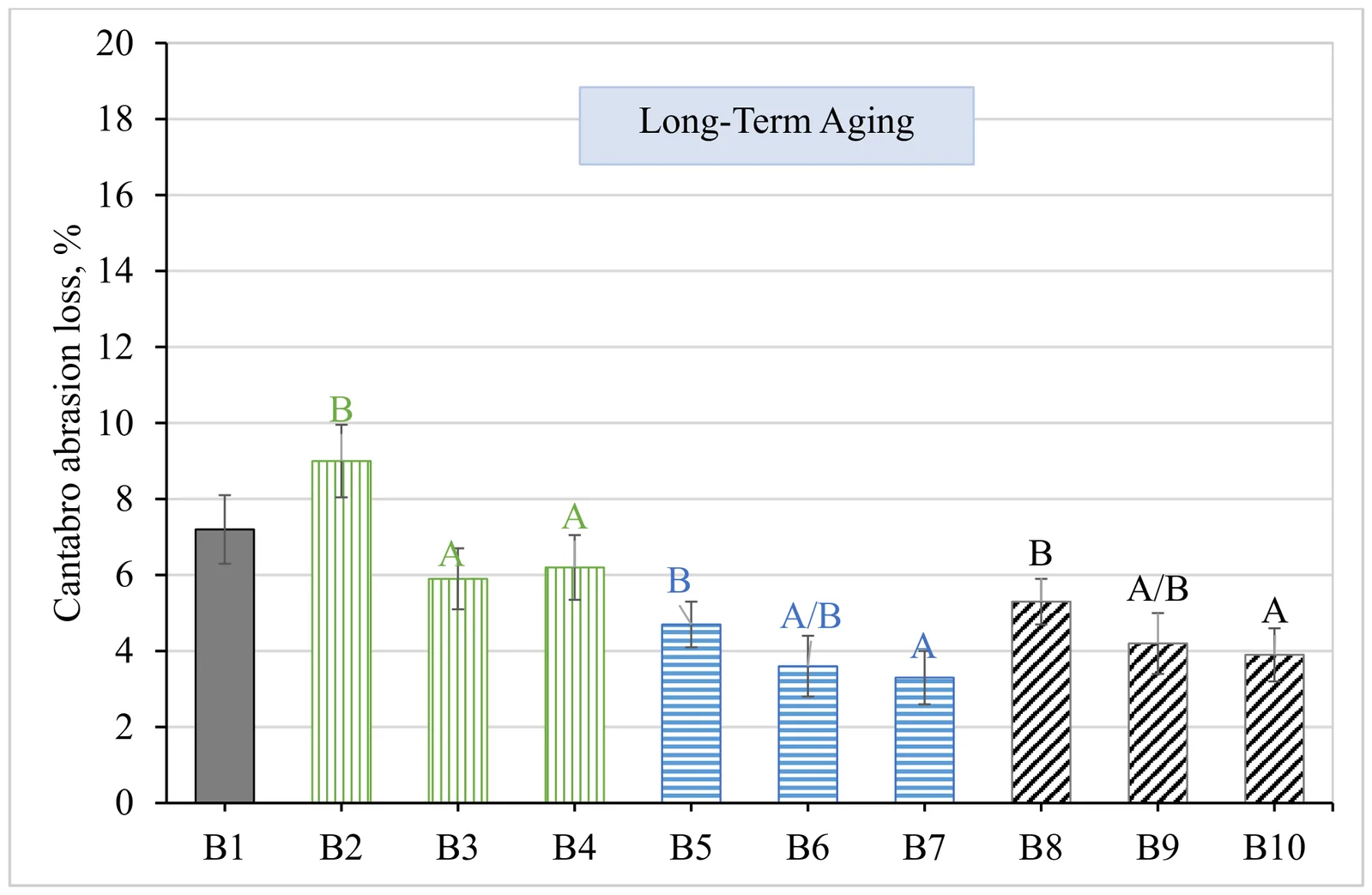
Cantabro abrasion loss test results—Long-term aging. Notes: Green letters represent RA1 statistical group, blue letters represent RA2 statistical group, and black letters represent RA3 statistical group.

Figure 8 presents the Cantabro abrasion loss (CAL) results for the evaluated mixtures after long-term aging (LTA). The coefficient of variation (CoV) ranged from 4% to 9%, with an average of 6.3%, indicating good testing repeatability. A lower Cantabro abrasion loss corresponds to greater resistance to raveling and improved mixture durability.

All evaluated mixtures exhibited Cantabro abrasion losses below 10%, indicating satisfactory abrasion resistance under the laboratory conditions used in this study. In general, abrasion loss decreased as recycling-agent dosage increased, demonstrating that greater binder softening improved the resistance of the aged mixtures to particle loss after long-term aging. This trend was observed for all three soybean-based recycling agents, with the greatest reductions generally occurring at the 4.0% dosage level. The improved CAL might be attributed to the rejuvenator’s ability to restore the aged binder’s properties, which in turn enhances the mixture’s resistance to disintegration [29,30,31].

The improved abrasion resistance is consistent with the corresponding increases in fracture resistance observed in the SCB and IDEAL-CT tests, indicating that the recycling agents enhanced the ability of the aged binder system to resist crack development and aggregate loss. However, the higher dosages that produced the lowest abrasion losses also resulted in reduced permanent-deformation resistance in the HWT test. Consequently, although the Cantabro abrasion loss test confirmed acceptable long-term durability for all mixtures, it was not selected as the controlling criterion for recycling-agent dosage selection. Instead, it served as a complementary durability indicator within the overall performance-based evaluation framework.

### 4.2. Statistical Analysis Summary for Scheffe’s Multiple-Comparison

Table 6 shows the summary for Scheffe’s multiple comparison of the 30% RAP mixtures (B2–B10).

### 4.3. Recycling-Agent Acceptable Dosage Range Determination

**Figure 9:**
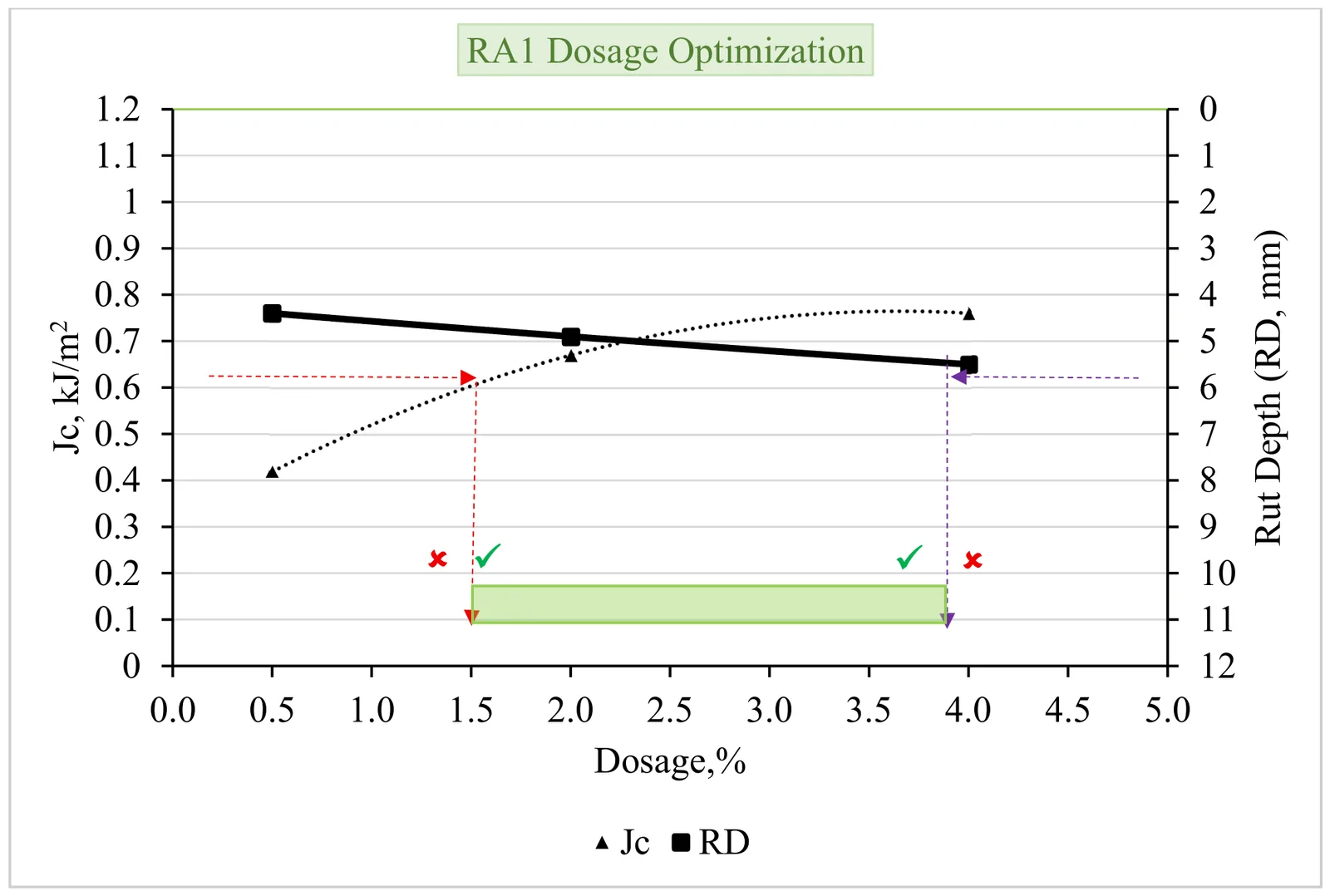
Acceptable dosage range determination for RA1 based on LTA SCB Jc and HWT rut depth. Notes: Green area represents the acceptable RA dosage that achieves cracking and rutting criteria.

**Figure 10:**
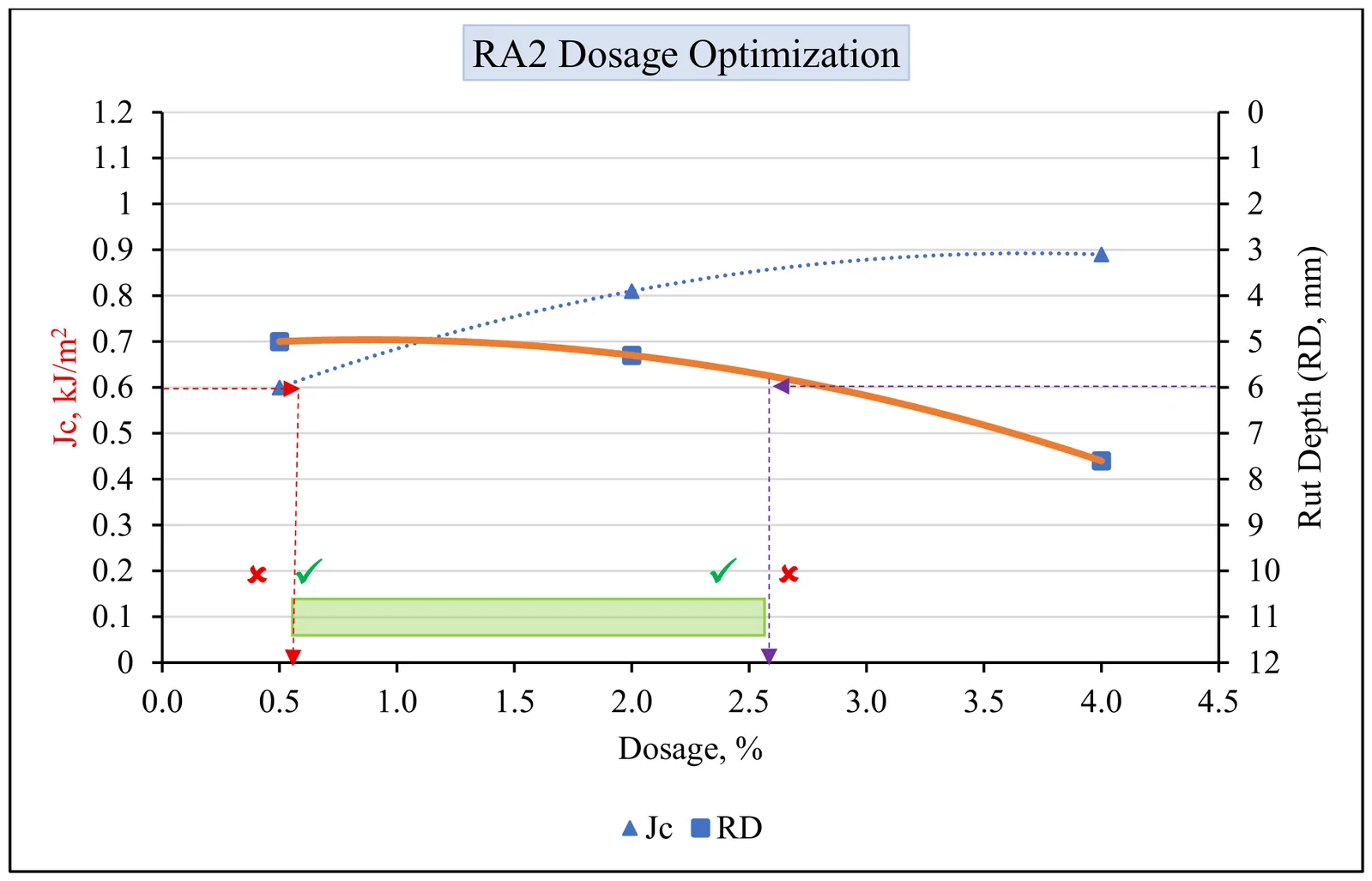
Acceptable dosage range determination for RA2 based on LTA SCB Jc and HWT rut depth. Notes: Green area represents the acceptable RA dosage that achieves cracking and rutting criteria.

**Figure 11:**
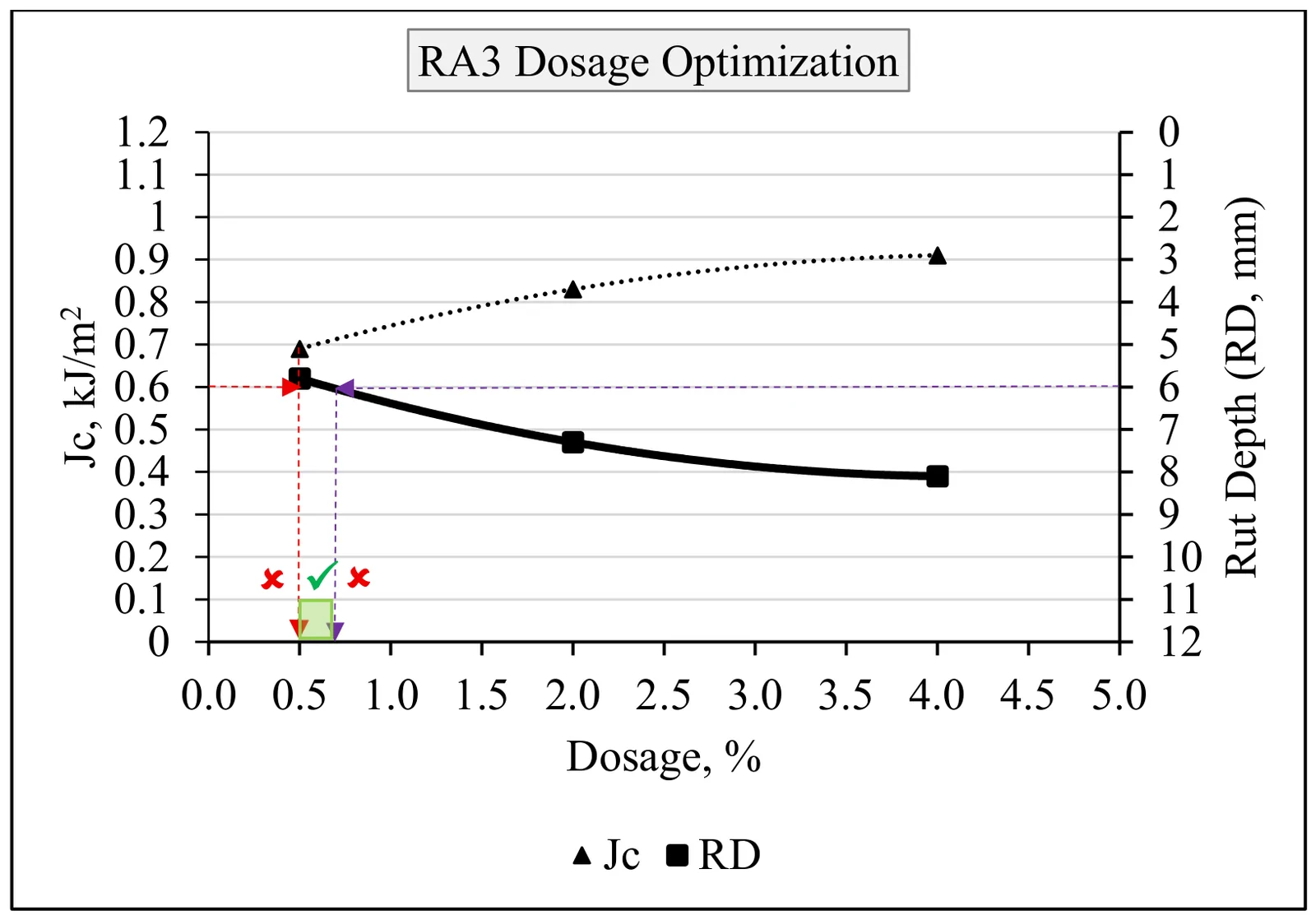
Acceptable dosage range determination for RA3 based on LTA SCB Jc and HWT rut depth. Notes: Green area represents the acceptable RA dosage that achieves cracking and rutting criteria.

Figure 9, Figure 10 and Figure 11 illustrate the performance-based procedure used to establish the acceptable dosage range for each soybean-based recycling agent by integrating the long-term aged SCB fracture criterion with the HWT rut-depth criterion. Recycling-agent dosage, expressed as a percentage by weight of binder, is shown on the x-axis. The left y-axis represents the SCB critical strain energy release rate (Jc), which characterizes cracking resistance, whereas the right y-axis represents HWT rut depth, which characterizes permanent-deformation resistance.

The green shaded areas in Figure 9, Figure 10 and Figure 11 indicate the proposed recycling-agent dosage ranges that satisfy the Louisiana DOTD BMD requirements, where the lower dosage boundary was determined by the cracking-resistance criterion and the upper dosage boundary was determined by the permanent-deformation criterion. These dosage ranges were determined directly from the intersection of the experimentally measured performance trends with the corresponding performance criteria in Figure 9, Figure 10 and Figure 11, using graphical interpolation between the evaluated dosage levels.

Increasing recycling-agent dosage consistently increased Jc while also increasing HWT rut depth, demonstrating the inherent tradeoff between improved cracking resistance and reduced permanent-deformation resistance. Consequently, the optimum dosage could not be established from either performance measure alone but required simultaneous consideration of both criteria.

For each recycling agent, the acceptable dosage range was defined as the dosage interval satisfying both the Louisiana DOTD Balanced Mix Design (BMD) requirements of Jc ≥ 0.6 kJ/m^2^ and HWT rut depth ≤ 6.0 mm. The lower dosage limit was governed by the minimum fracture-resistance requirement, whereas the upper dosage limit was controlled by the maximum allowable rut depth. The overlap between these two performance limits defined the acceptable dosage range.

Using this approach, the acceptable dosage ranges were determined to be 1.4–4.0% for RA1, 0.5–2.8% for RA2, and 0.5–0.7% for RA3, expressed as a percentage by weight of binder. These results clearly demonstrate the different softening efficiencies of the three soybean-based recycling agents. RA1 required relatively higher dosages to restore adequate fracture resistance because it produced the smallest reduction in binder stiffness. RA2 provided the broadest acceptable dosage range, indicating the most favorable balance between cracking resistance and permanent-deformation resistance. In contrast, RA3 produced the greatest increase in fracture resistance at low dosages but rapidly exceeded the rut-depth criterion as dosage increased because of its greater softening effect on the SBS-modified binder system.

Overall, the proposed framework provides a practical methodology for selecting recycling-agent dosage based on balanced mixture performance rather than binder softening or cracking performance alone. By integrating complementary cracking and rutting criteria, the approach enables optimization of soybean-based recycling agents for high-RAP, SBS-modified asphalt mixtures while satisfying the performance requirements of the Louisiana DOTD Balanced Mix Design (BMD) specification.

### 4.4. Summary of Results

Table 7 summarizes the relative laboratory performance of the 30% RAP mixtures (B2–B10) compared with the SBS-modified control mixture (B1). The symbols “+”, “=”, and “–” denote better, similar, and lower performance, respectively.

The experimental results consistently demonstrated that the type and dosage of soybean-based recycling agents governed the balance between cracking resistance and permanent-deformation resistance. Increasing recycling-agent dosage generally improved fracture resistance, as reflected by higher long-term aged SCB Jc values and increased IDEAL-CT indices, but simultaneously increased rut-depth susceptibility because of progressive binder softening. The relative softening effectiveness of the evaluated recycling agents consistently followed the order RA3 > RA2 > RA1, as confirmed by the binder characterization results (Table 2 and Table 3), the HWT rut-depth measurements, and the fracture-resistance tests.

The freeze–thaw-conditioned HWT results showed the same relative ranking of recycling-agent performance as the standard HWT test, indicating that moisture conditioning increased rut depth without changing the relative influence of recycling-agent type. The increase in rut depth following freeze–thaw conditioning was attributed primarily to reduced resistance to permanent deformation rather than moisture-induced stripping.

All mixtures exhibited satisfactory long-term durability, with Cantabro abrasion losses below 10%, indicating that abrasion resistance did not limit the use of any of the evaluated recycling agents. Overall, the laboratory results demonstrated a clear tradeoff between enhanced cracking resistance and reduced permanent-deformation resistance as recycling-agent dosage increased. Consequently, the acceptable recycling-agent dosage ranges were established by combining the long-term aged SCB Jc criterion with the standard HWT rut-depth criterion, providing a performance-based framework for optimizing the soybean-based recycling-agent dosage in SBS-modified asphalt mixtures containing 30% RAP.

## 5. Conclusions

This study evaluated the influence of three soybean-based recycling agents (RA1, RA2, and RA3) and their dosages on the rheological properties of asphalt binders and the engineering performance of SBS-modified asphalt mixtures containing 30% reclaimed asphalt pavement (RAP). A performance-based framework was developed to establish acceptable recycling-agent dosage ranges by integrating permanent-deformation and cracking-resistance criteria. Based on the materials, mixture design, and laboratory testing conditions used in this study, the following conclusions are drawn:

- Soybean-based recycling-agent type and dosage significantly influenced binder rheology and the resulting asphalt-mixture performance. Based on binder rheological characterization and asphalt-mixture performance testing, the relative softening effectiveness of the evaluated recycling agents followed the order RA3 > RA2 > RA1.
- Increasing recycling-agent dosage produced a consistent tradeoff between cracking resistance and permanent-deformation resistance. Higher dosages generally improved fracture resistance but increased rutting susceptibility, demonstrating that dosage selection should balance both performance mechanisms.
- The SCB and HWT tests were the primary performance indicators for establishing acceptable recycling-agent dosage ranges. IDEAL-CT, IDEAL-RT, freeze–thaw HWT, and Cantabro abrasion loss tests provided complementary validation of the observed performance trends.
- The proposed performance-based framework successfully established agent-specific acceptable dosage ranges for 30% RAP asphalt mixtures. Based on the combined SCB and HWT criteria, the acceptable dosage ranges were 1.4–4.0% for RA1, 0.5–2.8% for RA2, and 0.5–0.7% for RA3 (by weight of binder).
- Among the evaluated recycling agents, RA2 provided the broadest acceptable dosage range, indicating the most favorable balance between cracking resistance and permanent-deformation resistance. The proposed framework provides a practical approach for selecting the soybean-based recycling-agent type and dosage in high-RAP asphalt mixtures using balanced mix design principles.

The proposed framework was developed using a single RAP content (30%), one aggregate gradation, one virgin binder source, and the evaluated recycling agents. The reported dosage ranges were obtained by interpolation between the evaluated dosage levels (0.5%, 2.0%, and 4.0%) and should therefore be interpreted within the scope and experimental resolution of this study. Future research should evaluate the framework using additional materials, RAP contents, and dosage levels.

## 6. Recommendations

Future research should validate the proposed dosage-selection framework through plant production, field construction, and accelerated pavement testing to confirm its applicability under field conditions. Additional studies should investigate the long-term durability and aging characteristics of soybean-based recycling agents in polymer-modified asphalt mixtures and evaluate their environmental and economic benefits through life-cycle assessment (LCA) and life-cycle cost analysis (LCCA). Extending the framework to other polymer-modified binders, RAP contents, and recycling-agent chemistries would further demonstrate its general applicability.

## Figures and Tables

**Table 1:** SBS-modified control binder and soybean recycling-agent-modified asphalt binders evaluated in this study.

ID	Binder	Additive	Dosage (% by Weight of Binder)
B1	PG 76-22	SBS	3.5
B2	PG 67-22	RA1	0.5
B3	PG 67-22	RA1	2.0
B4	PG 67-22	RA1	4.0
B5	PG 67-22	RA2	0.5
B6	PG 67-22	RA2	2.0
B7	PG 67-22	RA2	4.0
B8	PG 67-22	RA3	0.5
B9	PG 67-22	RA3	2.0
B10	PG 67-22	RA3	4.0

**Note:** PG = performance grade; SBS = styrene-butadiene-styrene; RA1, RA2, and RA3 = Recycling agents. The PG 67-22 binder used in mixtures B2–B10 has SBS modification by the manufacturer. No additional SBS was added during the laboratory preparation of these mixtures.

**Table 2:** Physical properties of the soybean-based recycling agents used in this study.

Property	Test Method *	Units	Value/Type		
			RA1	RA2	RA3
Kinematic Viscosity @ 60 °C	RTM–110	cSt	80	25	20
Flash Point, Cleveland Open Cup	RTM–150	°F	>500	>430	>435
Flash Point, Pensky-Marten’s Closed Cup	RTM–151	°F	≥410	≥385	≥390
Viscosity Ratio	ASTM D2170 [13]		1.3	1.1	1.1
Weight Change	ASTM D2872 [14]	%	0.5	0.5	2.5

**Note:** * Testing done by the manufacturer; RTM: Resin Transfer Molding; RA: Recycling Agent.

**Table 3:** Asphalt binder performance grading test results for the SBS-modified control binder and recycling-agent-modified binders.

Test		Spec [15]	B1	B2	B3	B4	B5	B6	B7	B8	B9	B10
Original Binder												
Rotational Viscosity, 135 °C, Pa·s		3.0^−^	2.8	4.3	3.3	1.9	2.5	1.9	1.7	2.4	1.8	1.5
DSR, G*/sin(δ), kPa	76 °C	1.00^+^	2.58	6.61	3.28	1.82	2.27	1.73	1.58	2.06	1.88	1.61
	82 °C	1.00^+^	1.51	3.25	1.54	0.94	1.19	0.91	0.89	1.09	0.94	0.78
	88 °C	1.00^+^	0.92	1.61	0.74	NA	0.64	NA	NA	0.60	NA	NA
RTFO												
DSR, G*/sin(δ), kPa	76 °C	2.20^+^	2.95	NA	8.67	5.61	NA	NA	NA	NA	NA	NA
	82 °C	2.20^+^	1.76	NA	4.39	2.88	3.35	2.91	2.51	3.21	2.97	2.31
	88 °C	2.20^+^	NA	4.71	2.22	1.44	1.72	1.45	1.27	1.45	1.28	1.09
	94 °C	2.20^+^	NA	2.30	1.08	NA	NA	NA	NA	NA	NA	NA
	100 °C	2.20^+^	NA	1.17	NA	NA	NA	NA	NA	NA	NA	NA
RTFO + PAV												
DSR, 25.0 °C, G*·sin(δ), kPa, 5000^−^ for PG 67-22; 6000^−^ for PG 76-22M		5000^−^ 6000^−^	3660	10,713	8253	7660	9014	7120	6430	8106	6402	5716
BBR, Creep Stiffness, MPa, −6 °C		300^−^	NA	157	134	120	148	117	NA	131	98	NA
BBR, m-value, −6 °C		0.300^+^	NA	0.325	0.352	0.358	0.363	0.397	NA	0.372	0.384	NA
BBR, Creep Stiffness, MPa, −12 °C		300^−^	181	318	273	250	292	248	221	257	198	167
BBR, m-value, −12 °C		0.300^+^	0.344	0.250	0.278	0.290	0.272	0.294	0.308	0.281	0.292	0.312
BBR, Creep Stiffness, MPa, −18 °C		300^−^	380	NA	NA	NA	NA	NA	438	NA	NA	328
BBR, m-value, −18 °C		0.300^+^	0.255	NA	NA	NA	NA	NA	0.216	NA	NA	0.222
Performance Grade			PG 76-22	PG 88-16	PG 82-16	PG 76-16	PG 82-16	PG 76-16	PG 76-22	PG 82-16	PG 76-16	PG 76-22

**Note:** DSR = dynamic shear rheometer; BBR = bending beam rheometer; PG = performance grade; RTFO = rolling thin film oven; PAV = pressure aging vessel; NA = not available.

**Table 4:** Job mix formulas for the asphalt mixtures studied.

Parameter		B1	B2–10	DOTD Specs [12]
Asphalt Binder System		PG 76-22	RAs in PG 67-22	NA
Aggregate Blend	Limestone #78, %	52.5	39.0	NA
	Limestone #11, %	42.0	21.0	NA
	Limestone #9, %	5.5	9.9	NA
RAP Content, %		0.0	30.0	≤15.0
RBR		0.0	0.29	NA
P_b_, %		5.4	5.5	NA
RAP P_b_, %		0.0	5.2	NA
Number of Gyrations in SGC	Ni	7	7	7
	Nd	65	65	65
	Nf	105	105	105
G_se_		2.642	2.627	NA
G_mm_		2.482	2.452	NA
Design volumetric properties	%Gmm, Ni	87.1	88.4	≤89
	%Gmm, Nf	97.3	97.8	≤98
	VTM, %	3.6	3.4	2.5–4.5
	VMA, %	13.7	13.8	≥13.5
	VFA, %	74.4	77.2	69–80
	VFA, %	74.4	77.2	69–80
Effective P_b_, %		4.5	4.6	±0.2

**Note:** PG: Performance grade; RAs: Recycling agents; RBR: Recycled binder ratio; Ni: Initial number of gyrations; Nd: Design number of gyrations; Nf: Final number of gyrations; SGC: Superpave gyratory compactor; VTM; Voids in the total mix; VMA: Volume of mineral aggregates; VFA: Voids filled with asphalt; P_b_: Asphalt content by weight of mix (%); G_mm_: Theoretical maximum specific gravity of asphalt mixture; G_se_: Effective specific gravity of the aggregate; D:B: Ratio of dust to effective asphalt binder; N/A: Not applicable.

**Table 5:** List of physical and mechanical tests conducted on asphalt mixtures.

Test Designation	Aging	Testing Temperature (°C)	No. of Replicates/Sample Dimension: Dia. (mm) × Height (mm)	Engineering Property/Performance Indicator	Protocol/Standard
HWT	STA	50	4/D150 × H60	Permanent-Deformation Resistance	AASHTO T324 [19]
FT+HWT	STA	50	4/D150 × H60	Moisture Damage Resistance	Modified AASHTO T283 [20] AASHTO T324 [19]
SCB	STA	25	4/D150 × H57	Fracture Resistance	ASTM D8044 [21]
SCB	LTA	25	4/D150 × H57	Fracture Resistance	ASTM D8044 [21]
Cantabro Abrasion Loss	LTA	25	3/D150 × H115	Durability	AASHTO T401 [22]
IDEAL-RT	STA	50	3/D150 × H62	Permanent-Deformation Resistance	ASTM D8360 [23]
IDEAL-CT	LTA	25	3/D150 × H62	Fracture Resistance	ASTM D8225 [24]

**Note:** STA = short-term aging; LTA = long-term aging, 120 h at 85 °C; HWT = Hamburg Wheel Tracking test; FT = freeze–thaw conditioning; SCB = Semi-Circular Bend test; IDEAL-RT = IDEAL rutting test; IDEAL-CT = IDEAL cracking test.

**Table 6:** Scheffe’s multiple-comparison summary.

Test	Comparison	F_critical_	df_between groups_	df_within groups_	F_test_	*p*-Value	Significance
HWT-No FT	B2–B3	10.3	2	6	1.1	0.604	No
	B2–B4				11.1	0.043	Yes
	B3–B4				1.9	0.438	No
	B5–B6	10.3	2	6	1.8	0.455	No
	B5–B7				11.8	0.038	Yes
	B6–B7				11.3	0.042	Yes
	B8–B9	10.3	2	6	10.9	0.045	Yes
	B8–B10				11.3	0.042	Yes
	B9–B10				3.3	0.269	No
RT Index	B2–B3	10.3	2	6	1.2	0.579	No
	B2–B4				11.6	0.040	Yes
	B3–B4				0.8	0.690	No
	B5–B6	10.3	2	6	2.3	0.378	No
	B5–B7				12.1	0.036	Yes
	B6–B7				11.4	0.041	Yes
	B8–B9	10.3	2	6	11.6	0.040	Yes
	B8–B10				12.1	0.036	Yes
	B9–B10				2.2	0.392	No
HWT-FT	B2–B3	10.3	2	6	1.3	0.555	No
	B2–B4				11.1	0.043	Yes
	B3–B4				1.2	0.579	No
	B5–B6	10.3	2	6	1.4	0.533	No
	B5–B7				11.9	0.038	Yes
	B6–B7				10.9	0.045	Yes
	B8–B9	10.3	2	6	12.2	0.036	Yes
	B8–B10				12.7	0.033	Yes
	B9–B10				1.2	0.579	No
SCB-STA	B2–B3	10.3	2	6	11.1	0.043	Yes
	B2–B4				11.9	0.038	Yes
	B3–B4				1.2	0.579	No
	B5–B6	10.3	2	6	11.3	0.042	Yes
	B5–B7				12.2	0.036	Yes
	B6–B7				0.8	0.687	No
	B8–B9	10.3	2	6	10.9	0.045	Yes
	B8–B10				11.6	0.040	Yes
	B9–B10				1.4	0.533	No
SCB-LTA	B2–B3	10.3	2	6	11.2	0.042	Yes
	B2–B4				11.9	0.038	Yes
	B3–B4				2.1	0.406	No
	B5–B6	10.3	2	6	1.6	0.492	No
	B5–B7				12.6	0.034	Yes
	B6–B7				1.1	0.604	No
	B8–B9	10.3	2	6	1.3	0.555	No
	B8–B10				11.6	0.040	Yes
	B9–B10				1.7	0.473	No
CT Index	B2–B3	10.3	2	6	1.1	0.604	No
	B2–B4				1.2	0.579	No
	B3–B4				0.8	0.687	No
	B5–B6	10.3	2	6	0.9	0.658	No
	B5–B7				11.8	0.038	Yes
	B6–B7				1.4	0.533	No
	B8–B9	10.3	2	6	0.9	0.658	No
	B8–B10				1.1	0.604	No
	B9–B10				0.8	0.687	No
CAL	B2–B3	10.3	2	6	12.7	0.033	Yes
	B2–B4				10.8	0.046	Yes
	B3–B4				0.1	0.952	No
	B5–B6	10.3	2	6	2.4	0.364	No
	B5–B7				11.2	0.042	Yes
	B6–B7				8.1	0.077	No
	B8–B9	10.3	2	6	1.8	0.455	No
	B8–B10				12.2	0.036	Yes
	B9–B10				4.6	0.181	No

**Note:** STA = short-term aging; LTA = long-term aging, 120 h at 85 °C; HWT = Hamburg Wheel Tracking test; FT = freeze–thaw conditioning; SCB = Semi-Circular Bend test; IDEAL-RT = IDEAL rutting test; IDEAL-CT = IDEAL cracking test; df: degree of freedom.

**Table 7:** Relative performance of 30% RAP mixtures compared with the SBS-modified control mixture (B1).

Test	Engineering Property	Parameter	B2	B3	B4	B5	B6	B7	B8	B9	B10
HWT	Permanent-Deformation Resistance	Rut depths @ 20,000 Passes	=	=	-	=	-	-	-	-	-
FT+HWT	Moisture Susceptibility	Rut depths @ 20,000 Passes	=	-	-	-	-	-	-	-	-
SCB	Fracture Resistance	Jc	-	=	+	=	+	+	=	+	+
Cantabro Abrasion Loss	Durability	Abrasion Loss	=	=	=	+	+	+	+	+	+
IDEAL-RT	Permanent-Deformation Resistance	RT Index	=	=	=	+	+	=	+	=	=
IDEAL-CT	Fracture Resistance	CT Index	-	-	-	-	-	-	-	-	=

**Note:** HWT: Hamburg wheel tracking; SCB: Semi-circular bending; Jc: Critical strain energy release rate; FT: Freeze–thaw conditioning; +: Statistically better performance than the control mixture; -: Statistically lower performance than the control mixture; =: Statistically similar performance with the control mixture.

## Data Availability

The original contributions presented in this study are included in the article. Further inquiries can be directed to the corresponding author.
